# A Multimodal Biomedical Sensing Approach for Muscle Activation Onset Detection

**DOI:** 10.3390/s26061907

**Published:** 2026-03-18

**Authors:** Qiang Chen, Haofei Li, Zhe Xiang, Moxian Lin, Yinfei Yi, Haoran Tang, Yan Zhan

**Affiliations:** 1 Department of Physical Education and Military Affairs, China Jiliang University, Hangzhou 310018, China; 2 National School of Development, Peking University, Beijing 100871, China; 3 China Agricultural University, Beijing 100083, China; 4 Artificial Intelligence Research Institute, Tsinghua University, Beijing 100084, China

**Keywords:** medical rehabilitation monitoring, optical imaging, multimodal sensor fusion, biomedical sensing, lightweight temporal attention

## Abstract

Muscle onset detection is a fundamental problem in electromyography signal analysis, human–machine interaction, and rehabilitation assessment. In medical and biomedical applications, slow muscle activation onset processes are widely encountered in scenarios such as rehabilitation training, postural regulation, and fine motor control. Such processes are typically characterized by slowly varying amplitudes, long temporal durations, and high susceptibility to noise interference, which poses significant challenges for accurate identification of onset timing. To address these issues, a lightweight temporal attention method for slow muscle activation onset detection is proposed and systematically validated under multimodal experimental settings. The proposed method takes surface electromyography signals as the primary input, while synchronously acquired optical motion image data are incorporated into the experimental design and result analysis, thereby aligning with the common joint use of optical imaging and physiological signals in medical and biomedical research. From a methodological perspective, the proposed framework is composed of lightweight temporal feature encoding, a slow activation-aware temporal attention mechanism, and noise suppression with stable decision strategies. Under the constraint of low computational complexity, the ability to model progressive activation signals is effectively enhanced. Experiments are conducted on a dataset containing multiple types of slow activation movements, and model performance is evaluated using five-fold cross-validation. The results demonstrate that under regular signal-to-noise ratio conditions, the proposed method significantly outperforms traditional threshold-based approaches, classical machine learning models, and several deep learning baselines in terms of onset detection accuracy, recall, and precision. Specifically, onset detection accuracy reaches approximately 92%, recall is around 90%, and precision is approximately 93%. Meanwhile, the average onset detection error and detection delay are reduced to about 41ms and 28ms, respectively, with the false positive rate controlled at approximately 2.2%. Stable performance is further maintained under different noise levels and cross-subject settings, indicating strong robustness and generalization capability.

## 1. Introduction

Muscle onset detection is a fundamental problem in electromyography signal analysis and plays an essential role in motion control analysis, human–machine interaction, rehabilitation assessment, and wearable intelligent devices [1]. Accurate identification of the transition from resting to activated muscle states is a prerequisite for understanding motor intention, evaluating motor function, and enabling real-time control and feedback regulation [2]. In many practical scenarios, including rehabilitation medicine, elderly assistance, and fine motor tasks, muscle activation often exhibits slow and continuous characteristics, with small amplitude variations and long durations, which are commonly referred to as slow muscle activation onset processes [3]. Unlike fast activation—such as an explosive athletic sprint or a sudden reflex jerk characterized by high-frequency bursts and steep amplitude slopes—slow activation is akin to a gentle, sustained postural adjustment or a delicate fine-motor grasp, where signal energy gradually accumulates over hundreds of milliseconds. Compared with rapid contractions, slow activation better reflects natural human motor control patterns and is therefore critical for improving interaction naturalness and assessment accuracy [4]. However, such signals lack clear abrupt changes in both temporal and amplitude domains and are highly susceptible to background noise, inter-subject variability, and electrode drift, making reliable onset detection particularly challenging [5]. Traditional muscle onset detection approaches mainly rely on signal amplitude or energy variations, including fixed and adaptive threshold methods, sliding window statistics, and empirically designed energy operators [6], alongside classical formulations such as change-point detection algorithms, wavelet transform analysis, Bayesian inference models, and the Teager-Kaiser energy operator. These methods assume that muscle activation leads to significant changes in signal statistics and thus can perform stably under high-amplitude and rapid activation conditions [7]. Under slow activation, however, electromyography signals typically increase gradually with weak amplitude and low slope, making it difficult to achieve timely detection while maintaining low false alarm rates [8]. Threshold parameters are highly sensitive to noise and individual differences, often requiring repeated tuning, while local statistical measures are easily affected by transient noise. Consequently, under the low signal-to-noise ratio conditions typical of gradual muscle recruitment, studies have reported that traditional methods can incur detection delays well exceeding 100 milliseconds or exhibit false positive rates above 10% [9]. These limitations severely constrain the reliability and generalizability of traditional methods in slow activation scenarios [10].

With advances in deep learning for time-series modeling, convolutional neural networks, recurrent neural networks, and temporal convolutional networks have been increasingly applied to electromyography analysis [11]. Transformer models and their variants have further improved performance in complex electromyography pattern recognition and motor intention decoding tasks [12]. Nevertheless, most existing deep learning approaches rely on deep architectures and large parameter scales, leading to high computational complexity and inference latency [13], which are difficult to accommodate in wearable and embedded systems with limited resources [14]. For slow activation onset detection requiring long-term continuous monitoring, such high-complexity models introduce unnecessary redundancy and degrade real-time performance and stability [15]. Recent studies have explored lightweight attention-based designs, such as multiscale convolutional attention with transfer learning [16], channel activation attention mechanisms [17], spatio-temporal attention graph convolutional networks [18], and dual-attention temporal convolutional networks [19], demonstrating the potential of attention mechanisms under constrained resources. To address the challenges of weak signal variation, long temporal duration, and constrained deployment conditions in slow muscle activation onset detection, a lightweight temporal attention-based method is proposed. The architecture follows principles of lightweight modeling and deployability, combining shallow temporal feature encoding with an attention mechanism specifically tailored to slow activation characteristics. Key temporal segments are adaptively weighted to amplify weak progressive changes, while constrained attention structures reduce computational redundancy and noise sensitivity. This design enables favorable real-time performance and robustness under resource-limited conditions. The main contributions of this study are summarized as follows:

1. The fundamental differences between slow muscle activation onset detection and conventional onset detection are systematically analyzed from an application-oriented perspective, and key technical bottlenecks are identified.
2. A lightweight, slow activation-aware temporal attention network is proposed that integrates tailored noise suppression and stable decision strategies. This unified architecture effectively models long-term dependencies, amplifies weak progressive changes, and improves robustness across subjects and noise conditions without relying on computationally heavy deep architectures.
3. Comprehensive comparative experiments and ablation studies validate the advantages of the proposed method in terms of detection accuracy, response latency, and computational efficiency over existing paradigms.

## 2. Related Work

### 2.1. Muscle Onset Detection and Slow Activation Research

The primary goal of muscle onset detection is to identify the time point at which muscles transition from a resting to an activated state within continuous electromyography signals, which essentially corresponds to capturing changes in signal statistics or dynamic behaviors [20]. From a methodological perspective, traditional approaches can be fundamentally categorized as event-based detection paradigms. In these frameworks, early studies were mainly based on signal processing and statistical analysis, where variations in signal amplitude, energy, or local statistics were used to determine onset timing [21,22]. These methods rely on the assumption that muscle activation induces abrupt changes in signal distribution, allowing onset events to be detected once predefined thresholds or statistical criteria are exceeded [23]. Event-based detection inherently relies on the presence of sharp signal gradients or transient bursts. While this assumption generally holds under rapid or high-intensity activation, slow activation processes are characterized by low-amplitude, gradually evolving patterns, in which activation-related changes are easily masked by background noise [24]. Therefore, slow activation scenarios necessitate a process-based detection paradigm that continuously models cumulative temporal evolution rather than searching for isolated trigger points. Consequently, traditional threshold-based or local statistical approaches often fail to reliably distinguish true activation from noise-induced fluctuations in such scenarios. To alleviate these issues, several extensions have been proposed, including adaptive thresholds, dynamic window strategies, and multi-feature fusion to improve sensitivity to weak activations [25]. Although these methods partially reduce dependence on fixed parameters, they still rely heavily on handcrafted rules and empirical assumptions, making them sensitive to noise levels, electrode placement, and inter-subject variability [26]. During slow activation, the temporal scale of signal evolution often overlaps with that of noise disturbances, which further complicates robust parameter tuning [27]. More recent efforts have introduced refined time- or frequency-domain features to characterize progressive electromyography activity, yet these approaches remain largely confined to feature engineering and lack systematic modeling of slow activation dynamics, resulting in limited robustness across subjects and tasks [28].

### 2.2. Deep Learning for Electromyography Time-Series Modeling

With the development of machine learning, data-driven approaches have been increasingly applied to muscle onset detection and electromyography analysis [29]. Deep learning models enable automatic learning of hierarchical representations through end-to-end training and show clear advantages in capturing nonlinear temporal patterns [30]. Convolutional neural networks extract local temporal structures via local receptive fields, while recurrent architectures model temporal dependencies through hidden state propagation and have been widely used in motion recognition and intention decoding tasks [31]. However, when applied to slow activation scenarios, these standard architectures exhibit distinct suboptimal characteristics. Conventional convolutional networks often struggle because their localized receptive fields fail to capture the prolonged, gradual amplitude variations that characterize slow onsets. Similarly, recurrent architectures are prone to noise accumulation and gradient instability over the extended temporal monitoring windows required to capture slow activation processes. Temporal convolutional networks further extend receptive fields using dilated convolutions while maintaining parallel computation, offering an efficient solution for long-sequence modeling [32]. These models have improved overall electromyography analysis performance and opened new possibilities for slow activation onset detection [33]. Attention-based models, particularly transformer architectures, have recently gained prominence in time-series modeling [34]. By explicitly modeling correlations among time steps, self-attention overcomes limitations in capturing long-term dependencies and has achieved strong performance in complex temporal tasks [35]. Transformer-based methods have been explored for electromyography modeling, where global attention highlights key temporal segments to enhance sensitivity to subtle patterns [36]. However, these models typically involve high-dimensional representations and parameter scales, leading to quadratic computational complexity with respect to sequence length, which limits real-time performance and energy efficiency [37]. For slow activation detection, where changes are weak and localized but monitoring is long-term, full attention mechanisms often introduce redundancy and increased noise sensitivity [38].

### 2.3. Lightweight Models and Attention Mechanisms

As wearable and embedded platforms become central to electromyography applications, lightweight model design has emerged as a critical research direction [39]. Lightweight networks aim to reduce model size and inference cost through structural simplification or parameter compression while retaining essential modeling capacity [40]. Prior studies indicate that task-oriented architectural customization is more effective for stable deployment under resource constraints than directly adopting generic deep models [41]. Nevertheless, most lightweight approaches focus on classification or regression and provide limited capability for selecting informative temporal segments, which restricts their applicability to slow activation detection involving weak variations over long durations [42]. Attention mechanisms offer a promising solution by assigning temporal weights to emphasize task-relevant segments, thereby improving effectiveness without substantially increasing parameters [43,44]. However, conventional attention schemes often rely on global modeling, resulting in higher computational and memory costs, and may assign excessive weights to non-informative regions in noisy physiological signals [45]. How to design constrained and efficient attention mechanisms that preserve sensitivity to weak, progressive activations while satisfying real-time and stability requirements remains an open challenge [46].

To provide a clearer overview of the existing literature and better structure the comparisons discussed above, Table 1 summarizes the primary modeling paradigms and explicitly highlights their key limitations when applied to the slow muscle activation onset detection task.

## 3. Materials and Method

### 3.1. Data Collection

The dataset used in this study was obtained from a multimodal human motion and electromyography synchronous acquisition experiment. The participants consisted of 30 healthy volunteers (15 males, 15 females; age range: 22–35 years) recruited through convenience sampling from the local university community. The inclusion criteria required participants to be within the specified age range and to have normal motor function. The exclusion criteria included a history of neuromuscular diseases, recent musculoskeletal injuries, or the current use of medications affecting motor performance. The study protocol was formally approved by the local Ethics Committee and the Institutional Review Board (IRB). Prior to data acquisition, action demonstrations and training instructions were provided to all participants, and written informed consent was obtained. To further ensure representativeness with respect to inter-subject variability and test the model’s robustness across different sensor configurations, a portion of publicly available online data was combined with our proprietary dataset, as shown in Table 2. The rationale for this combination was to encompass a wider diversity of physiological traits. Consistency in acquisition protocols was strictly maintained by selecting public datasets that utilized similar slow activation paradigms and standardizing the signals through re-sampling and uniform preprocessing. The data collection was conducted from March 2024 to May 2024. The experimental protocol was designed around slow muscle activation onset processes, requiring participants to perform voluntary contraction and relaxation of target muscle groups in a slow and controlled manner under resting conditions. The actions included low-speed joint flexion and extension, gradual force exertion during posture maintenance, and low-intensity control movements commonly encountered in rehabilitation training. Each participant completed multiple repeated trials, with each trial emphasizing a smooth transition from a fully resting state to an activated state to ensure the acquisition of representative slow activation onset signals.

**Figure 1:**
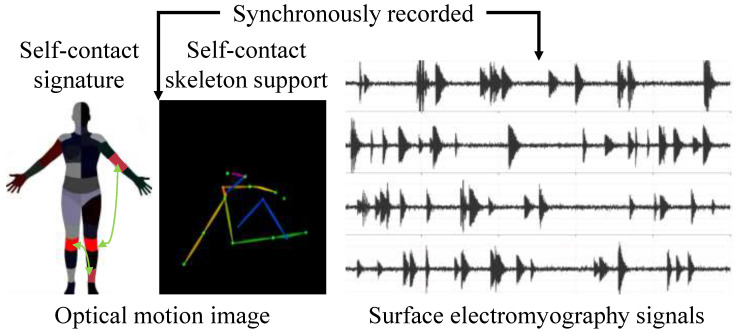
Schematic representation of optical motion images and corresponding surface electromyography signals. Volunteers performed instructed self-contact motions, while electrodes placed over the relevant muscle groups synchronously recorded the electromyographic activity.

Electromyography data were synchronously recorded using a multichannel surface electromyography acquisition system, as shown in Figure 1. Electrodes were attached to the surface of target muscle groups in accordance with standard muscle localization guidelines, and skin preparation and impedance inspection were conducted prior to acquisition to reduce contact noise and electrode drift. The sampling frequency of the electromyography signals was set to 1000 Hz to fully preserve the subtle yet continuous amplitude variations during slow activation phases. During acquisition, the raw electromyography signals were stored as continuous time series. Their signal characteristics were typically characterized by low signal-to-noise ratios, absence of obvious abrupt changes, and relatively long duration, which are consistent with the typical properties of slow muscle activation onset processes.

In addition to electromyography signals, human motion image data were synchronously collected to assist in analyzing the correspondence between muscle activation and external motion manifestations. The image data were acquired using fixed-view high-definition cameras, fully covering the entire execution process of each action. These data primarily captured changes in limb posture, gradual adjustments of joint angles, and subtle displacements occurring during the early stages of motion onset.

To establish accurate reference labels for model evaluation, a rigorous manual annotation procedure was implemented. The ground-truth onset was defined as the exact time point when the continuous electromyography signal exhibited the first sustained deviation from the baseline resting noise level, which was further cross-verified by identifying the initial visible movement in the synchronous video data. The annotation process was conducted independently by two experts with extensive experience in biomedical signal processing. To ensure labeling consistency and minimize subjective bias, an inter-rater reliability assessment was performed. The Intraclass Correlation Coefficient (ICC) was calculated between the two annotators, yielding a high level of agreement. In cases where the annotated onset times differed by more than 50 milliseconds, a third senior expert reviewed the specific trial to resolve the discrepancy and reach a final consensus, thereby guaranteeing the high quality of the ground-truth labels.

### 3.2. Data Preprocessing and Augmentation Strategy

Surface electromyography signals constitute a typical class of non-stationary physiological time series, whose acquisition process is easily affected by environmental noise, variations in electrode contact conditions, and inter-subject physiological differences. Particularly in slow muscle activation onset detection tasks, target signals usually manifest as low-amplitude and gradually varying progressive processes, where the statistical differences between activation-related components and background noise are not pronounced. This characteristic imposes higher requirements on the stability and robustness of subsequent modeling procedures. Therefore, appropriate preprocessing and task-oriented data augmentation applied to raw electromyography signals prior to model training are critical for improving detection accuracy and generalization capability.

From a signal processing perspective, the informative components of electromyography signals are mainly concentrated within specific frequency bands, whereas low-frequency drift and high-frequency interference generally do not convey discriminative information directly related to muscle activation. Accordingly, a fourth-order Butterworth band-pass filter with cutoff frequencies of 20 to 450 Hz is first applied to the raw electromyography signals to suppress the effects of direct current offset, motion artifacts, and high-frequency electromagnetic noise. Let the original single-channel electromyography signal be denoted as x(t), and the filtered signal can be expressed as

(1) $$x_{f}\left(t\right)=x\left(t\right)*h\left(t\right),$$

where h(t) represents the impulse response of the band-pass filter and ∗ denotes the convolution operation. By appropriately selecting the passband range of the filter, the primary electromyography activity components can be preserved while noise interference during slow activation phases is significantly reduced. The filtered signals exhibit improved stationarity in the time domain, providing a more reliable input foundation for subsequent feature extraction and temporal modeling.

After filtering, the substantial amplitude distribution differences across subjects must still be addressed. Due to variations in skin impedance, electrode placement, and individual muscle physiological characteristics, electromyography signals collected from different subjects may exhibit markedly different amplitude ranges even under identical task conditions. If unnormalized signals are directly fed into the model, a bias toward samples with larger amplitudes may be introduced during training, thereby reducing sensitivity to weak activation signals. To mitigate this issue, normalization is applied to the filtered electromyography signals to constrain their numerical distributions within a relatively consistent range. Let the filtered signal be $x_{f}\left(t\right)$, and its normalized form is defined as

(2) $$x_{n}\left(t\right)=\frac{x_{f}\left(t\right)-\mu }{\sigma },$$

where μ and σ denote the mean and standard deviation of the signal computed across the entire continuous recording trial for each subject, rather than on a local window-by-window basis. This global normalization strategy effectively eliminates amplitude scale differences and enables the model to focus on relative variation patterns rather than absolute amplitude values, which is particularly important for modeling subtle changes during slow activation onset phases without losing the macroscopic progressive amplitude trends.

Following filtering and normalization, a sliding window segmentation strategy is adopted to divide continuous electromyography signals into multiple local temporal segments, thereby facilitating subsequent temporal modeling. Let the normalized signal be $x_{n}\left(t\right)$; by applying a sliding window with length *L* and stride *S*, the *k*-th temporal segment can be obtained as

(3) $$x_{k}=\left[x_{n}\left(kS\right),x_{n}\left(kS+1\right),\ldots ,x_{n}\left(kS+L-1\right)\right].$$

Sliding window segmentation converts long time series into fixed-length input units, reducing training difficulty while preserving temporal continuity of the slow activation process. By appropriately selecting the window length *L* and stride *S*, a balance between temporal resolution and computational complexity can be achieved, enabling the model to capture progressive changes during slow activation without introducing excessive redundant computation.

Beyond basic preprocessing steps, lightweight data augmentation strategies are further introduced to address sample imbalance and the high proportion of weak activation samples commonly encountered in slow muscle activation onset detection tasks. Unlike complex augmentation techniques used in image or speech domains, the augmentation strategies adopted here are closely aligned with the physical characteristics of slow activation signals and are designed to introduce reasonable perturbations without disrupting temporal structure.

Amplitude perturbation serves as an effective means to simulate uncertainty in electromyography signal amplitudes. In practical acquisition scenarios, even for the same subject performing identical tasks, signal amplitudes may fluctuate due to factors such as fatigue level and changes in electrode contact conditions. To enhance robustness to such variations, random amplitude scaling is applied to sliding window segments. Let the original segment be $x_{k}$, and the amplitude-perturbed signal can be expressed as

(4) $${\tilde{x}}_{k}=\alpha x_{k},$$

where α is a scaling factor randomly sampled from a predefined range. With appropriate constraints on α, different amplitude conditions of slow activation processes can be simulated without altering the overall signal morphology, thereby improving the model’s adaptability to weak activation signals.

In slow activation scenarios, variations in temporal scale are also prominent. The duration required for muscles to transition from resting to activated states may differ substantially across subjects or task conditions. To model this phenomenon, a temporal scale perturbation strategy is introduced, in which signals are lightly stretched or compressed along the time axis. Let the original time index be *t*, and the transformed index is given by

(5) $$t^{\prime }=\beta t,$$

where β denotes the temporal scaling factor. When β>1, the signal is stretched along the time axis, simulating slower activation processes; when β<1, the signal is compressed, corresponding to relatively faster activation dynamics. By remapping the transformed signal to the original length through interpolation, diverse activation temporal patterns can be introduced while maintaining consistent input dimensions. Crucially, because temporal scaling alters the time axis, it is imperative to ensure that the ground-truth onset labels remain perfectly synchronized with the augmented signals. Therefore, whenever the temporal scale of the electromyography signal is modified by a factor of β, the corresponding ground-truth onset timestamps are strictly scaled by the exact same mathematical factor. This synchronized adjustment guarantees exact temporal alignment and prevents any label mismatch during the training phase. This augmentation strategy plays an important role in enhancing generalization to different slow activation rhythms.

It should be noted that all data augmentation strategies employed in this study are lightweight operations with negligible computational overhead and, thus, do not significantly increase overall training or inference costs. Moreover, these strategies do not rely on artificial noise distribution assumptions but instead model natural variations observed in practical electromyography acquisition, ensuring the physical plausibility of augmented samples. By integrating preprocessing and augmentation strategies, signal quality and sample diversity are effectively enhanced at the input level, providing a solid foundation for stable training of the proposed lightweight temporal attention model.

### 3.3. Proposed Method

#### 3.3.1. Overall

It is important to clarify the role of the multimodal data within the proposed framework. While the overall data acquisition and validation paradigm is inherently multimodal—utilizing synchronous optical motion signals to cross-verify and establish highly accurate ground-truth annotations for the subtle, early stages of slow muscle activation—the computational model itself is purposefully designed to rely exclusively on surface electromyography signals during inference. This design choice ensures that the detection model remains lightweight and easily deployable in real-world wearable scenarios where continuous optical tracking is impractical. After the required signal preprocessing and segmentation, the processed electromyography time-series data are fed into the proposed lightweight temporal-attention detection framework as model inputs. The overall method is constructed in an end-to-end manner, forming a complete and well-structured computational pipeline from input feature encoding and temporal relationship modeling to onset-time decision making. First, the segmented multi-channel electromyography sequences are delivered to the lightweight temporal feature encoding module, where shallow 1D convolutions are employed to model local variation patterns along the time axis and to extract discriminative low-dimensional feature representations while preserving the original temporal structure. The resulting representations not only reduce input dimensionality and suppress redundant noise components but also provide a more stable and compact temporal basis for subsequent modules. Next, the encoded features are forwarded to the slow activation-aware temporal attention module, where the importance of different temporal segments is adaptively assessed. A constrained and factorized attention structure is adopted to assign higher weights to potential slow activation onset regions, thereby effectively amplifying persistent yet low-amplitude variations in the feature space. Unlike global attention, high-complexity modeling over the entire sequence is avoided, and computation is concentrated on local temporal regions most relevant to onset decision making, achieving a balance between efficiency and modeling capacity. The attention-weighted temporal features are then passed to the onset decision module. At this stage, response consistency across consecutive time segments is jointly considered, and smoothing constraints together with stable decision rules are imposed on the attention-enhanced features to suppress the influence of instantaneous noise fluctuations on detection outcomes. Finally, a time-varying onset confidence response is produced, and the slow muscle activation onset time is determined once stability conditions are satisfied. Through the progressive coupling of these modules, a complete mapping from electromyography temporal patterns to reliable onset decisions is achieved, where lightweight and deployable design objectives are maintained while targeted modeling is performed for weak variations, long temporal spans, and high-noise characteristics in slow activation scenarios.

#### 3.3.2. Lightweight Temporal Feature Encoding

**Figure 2:**
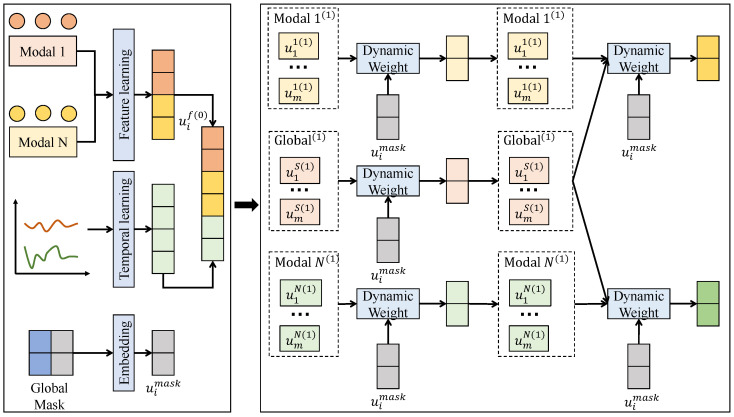
Illustration of the lightweight temporal feature encoding module, where shallow temporal encoding and task-adaptive mixture-of-experts weighting enable effective modeling and fusion of slow activation-related temporal features under low computational complexity.

The lightweight temporal feature encoding module serves as the first stage of the overall detection framework. Its objective is not to construct high-level abstract semantics but to stably extract fundamental temporal structures associated with slow activation onset under minimal computational cost. As shown in Figure 2, this module directly receives the multi-channel electromyography sequences after preprocessing and sliding-window segmentation. Let an input segment be denoted as $X\in R^{C\times T}$, where *C* represents the number of electromyography channels, *T* denotes the window length, and $X_{c,t}$ corresponds to the electromyography response of the *c*-th channel at time *t*. Considering that slow activation signals exhibit small-amplitude variations, smooth temporal evolution, and locally continuous patterns, shallow 1D convolutions are adopted to model the signal along the time axis, while higher-order nonlinear interactions across time or across channels are avoided to prevent unnecessary parameter expansion.

In implementation, temporal feature encoding is realized by one or two 1D convolution layers. For the single-layer convolution case, the computation can be formally written as

$$F_{k,t}=\sum_{c=1}^{C}\sum_{\tau =0}^{K-1}W_{k,c,\tau }\;X_{c,t-\tau }+b_{k},$$

where $W_{k,c,\tau }$ denotes the weight of the *k*-th convolution kernel on the *c*-th channel at temporal offset τ, *K* is the kernel width along the time dimension, $b_{k}$ is the bias term, and $F\in R^{C^{\prime }\times T}$ is the output feature map with $C^{\prime }$ channels, representing either compression or moderate expansion of feature channels. A small kernel width *K* is selected so that the receptive field concentrates on adjacent temporal segments, thereby highlighting continuous yet low-amplitude variations during slow activation. When two convolution layers are used, the second layer is designed as a lightweight mapping along the channel dimension, whose primary role is to stabilize feature distributions rather than to enlarge the temporal receptive field.

From a mathematical perspective, this shallow convolutional structure can be viewed as applying a set of learnable local time-domain filters to the raw electromyography sequence, where the outputs emphasize relative variations at short temporal scales instead of absolute amplitude magnitudes. This property is well aligned with slow activation onset detection, because slow activation does not rely on instantaneous amplitude peaks but is primarily characterized by progressive temporal trends. Moreover, due to the translation invariance of convolution along the time axis, the feature mapping $F_{:,t}$ follows a consistent transformation rule for any time position *t*, enabling uniform perception of local patterns before and after onset across different temporal locations.

In terms of module coupling, the output feature sequence *F* remains fully aligned along the time dimension and is directly fed into the slow activation-aware temporal attention mechanism. Since preliminary denoising and dimensionality reduction have already been performed in the original signal space during encoding, the attention module no longer needs to learn weight distributions from high-noise, high-dimensional inputs, which substantially reduces instability in attention weight estimation. Overall, the proposed design of shallow convolution, local modeling, and low-dimensional mapping explicitly encodes slow activation-related temporal structures into the feature space, providing stable, efficient, and deployment-oriented representations for subsequent attention weighting and onset decision making.

#### 3.3.3. Slow-Activation-Aware Temporal Attention Mechanism

**Figure 3:**
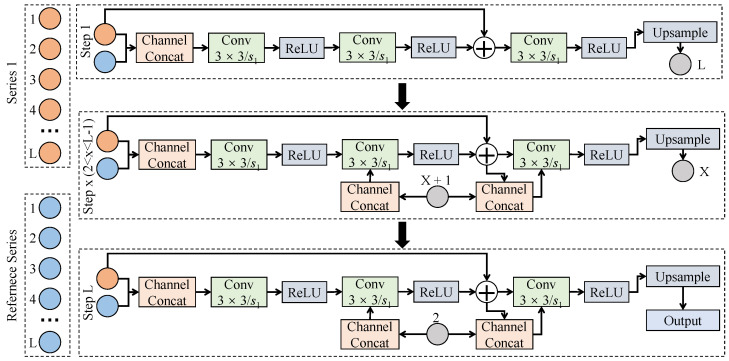
Illustration of the slow activation-aware temporal attention mechanism, where progressive interactions between neighborhood and reference time steps adaptively emphasize key temporal features associated with slow activation onset.

The slow-activation-aware temporal attention mechanism is designed to address the modeling challenge that key information is weak yet persistent in slow activation onset detection, and its design differs fundamentally from standard self-attention. Standard self-attention typically constructs global query–key–value mappings to explicitly model correlations between arbitrary time steps, where attention weights are computed with participation from all temporal positions. As a consequence, computational complexity grows quadratically with sequence length, and irrelevant segments may receive excessive weights in noisy physiological signals. In contrast, the proposed slow-activation-aware temporal attention does not aim at global correlation modeling. Instead, it is tailored to the characteristics of slow activation onset, namely, locally continuous, trend-dominant, and low-amplitude variations, by adopting a constrained receptive field and neighborhood-aware attention structure that models relationships only among adjacent or near-adjacent segments. Therefore, computational cost is reduced while sensitivity to progressive changes is enhanced. As shown in Figure 3, this attention mechanism takes the feature sequence produced by the lightweight temporal encoding module as input and performs progressive interaction between neighborhood inputs and reference inputs along the time axis, rather than performing a one-shot global reweighting over the entire sequence. To provide a more intuitive understanding for non-specialist readers, this process can be likened to a sliding magnifying glass. Instead of looking at the entire recording simultaneously to determine which parts are important, the proposed mechanism focuses strictly on a small, moving time window. It evaluates the current moment based solely on its immediate surroundings to decide if it is part of a slow, building muscle contraction, thereby effectively ignoring distant, irrelevant noise.

In terms of architecture, the slow activation-aware temporal attention mechanism is constructed by serially connecting attention units corresponding to *L* temporal steps, and each unit applies a shared-parameter shallow convolutional structure to model local relationships. Let the encoded input feature be $F\in R^{C\times T}$, where *C* is the channel number and *T* is the temporal length. For the *t*-th time position, neighborhood features and reference features are first concatenated along the channel dimension to form a local context representation

(6) $$Z_{t}=\mathrm{Concat}\;\left(F_{:,t-k:t+k},\;F_{:,t}\right),$$

where *k* is the neighborhood radius controlling the temporal receptive range. Then, $Z_{t}$ is transformed by two shared 1D convolution layers with kernel size 3×1 and stride 1. The first layer maps channels to $C_{a}=32$, and the second layer further compresses to $C_{b}=16$, with ReLU activation applied between layers. The transformed features are subsequently passed to an attention generation layer to obtain the attention weight at time *t*:

(7) $$\alpha_{t}=\sigma \;\left(W_{a}*Z_{t}+b_{a}\right),$$

where $W_{a}$ denotes a 1×1 convolution kernel and σ(·) is the Sigmoid function constraining weights to [0,1]. Finally, the attention weight is applied to the original feature to obtain the slow activation-enhanced representation:

(8) $${\tilde{F}}_{:,t}=\alpha_{t}\cdot F_{:,t}.$$

By repeating this procedure for all *t*, the attention-weighted feature sequence $\tilde{F}$ is obtained, where the temporal length remains unchanged while slow-activation-related segments are strengthened in the feature space.

It is important to clarify how this formulation fundamentally differs not only from global self-attention but also from other local or temporal attention variants. Many local attention mechanisms still compute dot-products between all elements within a restricted window, which maintains a quadratic complexity locally. Other temporal attention variants often rely on rigid temporal convolutions with fixed weights. In contrast, our approach adaptively generates a single scalar weight for each time step directly from its local context via lightweight convolutions, entirely avoiding the computational overhead of inner-product operations while specifically targeting the monotonic, gradual trends characteristic of slow activations. From a theoretical standpoint, the proposed mechanism can be interpreted as a constrained weighted smoothing operator, where weights are adaptively generated from local temporal context rather than determined by global inner-product relations. Since slow activation onset processes are temporally continuous, it follows that when activation trends are monotonic within local neighborhoods, the resulting attention weight sequence $\left\{\alpha_{t}\right\}$ satisfies an implicit smoothness constraint, avoiding abrupt oscillations between adjacent time segments. This property prevents overreaction to instantaneous noise and enables cumulative enhancement as activation trends gradually increase. The mechanism naturally complements the preceding lightweight temporal feature encoding module, where local variation patterns are stably extracted by encoding and then selectively amplified along the time axis by slow activation-aware attention. Through this coupled design, effective modeling of slow activation onset is achieved without introducing the high computational complexity of standard self-attention, thereby jointly supporting detection performance, computational efficiency, and practical deployability.

#### 3.3.4. Noise Suppression and Stable Decision Strategy

**Figure 4:**
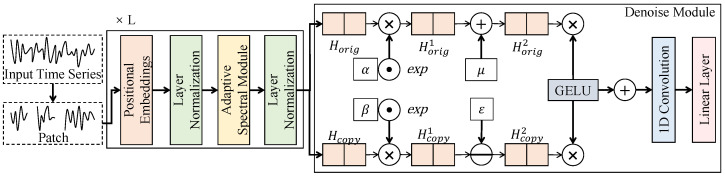
Illustration of the noise suppression and stable decision strategy, where spatiotemporal joint modeling and gated fusion impose smoothing constraints on slow activation onset responses while suppressing instantaneous noise interference.

The noise suppression and stable decision strategy is positioned at the end of the overall model, aiming to suppress instantaneous noise interference and local abnormal fluctuations commonly observed in electromyography signals while maintaining sensitivity to slow activation onset responses. As shown in Figure 4, this strategy is not implemented as a simple threshold-based post-processing step but is realized through end-to-end modeling that integrates an embedded spatiotemporal joint attention component with stable decision rules. Specifically, the output features from the slow-activation-aware temporal attention module are denoted as $\tilde{F}\in R^{C\times T}$, where *C* is the channel dimension and *T* is the temporal length. To simultaneously model channel-wise collaboration and temporal evolution continuity, a stacked 1D convolution and gated fusion structure is adopted to progressively refine representations along the time axis. The overall network consists of L=3 stable decision units, each maintaining an intermediate representation with channel width $C_{s}=16$. Local aggregation along the time dimension is performed using 3×1 1D convolutions, enabling temporal alignment to be preserved while redundant noise is gradually compressed.

Within each unit, the input features are first processed through a linear mapping and a nonlinear gating operation to form two parallel branches, denoted as $U_{t}$ and $V_{t}$, both aligned with the original features along the time axis. The stably fused representation $S_{t}$ is obtained by

(9) $$S_{t}=\phi \left(U_{t}\right)\cdot \psi \left(V_{t}\right),$$

where ϕ(·) and ψ(·) denote a smoothing activation function and a gating function, respectively, which control feature magnitude and suppress abrupt spikes. This structure is mathematically equivalent to applying an adaptive low-pass filtering operator to the temporal sequence, such that high-frequency instantaneous noise is progressively attenuated through multi-layer propagation while low-frequency variations persistently present during slow activation are retained. Since each unit operates within local temporal windows, the receptive field grows linearly with the number of layers, thereby imposing temporal consistency constraints without introducing global dependencies.

After spatiotemporal joint modeling, stable decision-making is performed by imposing a temporal consistency constraint on onset responses. Let the final temporal response be denoted as $y_{t}$, which can be interpreted as a time-varying slow activation confidence curve. To avoid false triggering induced by single-point noise, the decision is not based on an extreme value at a particular time but is instead determined by a continuity criterion over a temporal window Ω(t), expressed as

(10) $$\frac{1}{\left|\Omega (t)\right|}\sum_{\tau \in \Omega (t)}y_{\tau }>\theta ,$$

where θ is the stable decision threshold. The parameters governing this continuity criterion, specifically the decision threshold θ and the window size |Ω(t)|, were systematically determined through empirical grid search on the validation set. Once optimal values were identified, these parameters were kept strictly fixed across all subjects and tasks during the testing phase to ensure a rigorous evaluation of the model’s cross-subject generalization capabilities. Furthermore, compared to classical “N consecutive samples” rules—which strictly require the signal to exceed a threshold at every single time step within a window and are thus highly vulnerable to isolated noise dips that prematurely reset the detection counter—the proposed average-based continuity criterion offers significantly greater resilience. It naturally tolerates minor, instantaneous fluctuations within the window as long as the cumulative response remains strong. Under the assumption that the noise term is zero-mean or locally symmetric, its expected contribution within a sliding temporal window tends toward 0, whereas the slow activation signal exhibits persistence and yields an accumulated response that increases over time. Therefore, this criterion can distinguish true onset events from instantaneous noise perturbations in a probabilistic sense. This decision strategy complements the preceding slow-activation-aware temporal attention module, where attention highlights potential onset regions at the feature level and stable decision-making enforces temporal consistency at the decision level.

## 4. Results and Discussion

### 4.1. Experimental Configuration

#### 4.1.1. Hardware and Software Platform

The experiments were conducted on a general yet representative deep learning computing platform to validate the feasibility and engineering adaptability of the proposed method under non-high-end computational conditions. The hardware platform was equipped with an NVIDIA GeForce RTX 3090 GPU (24 GB VRAM) for model training and inference acceleration, providing sufficient memory capacity to support batch-parallel computation of multi-channel electromyography sequences. An Intel Core i9-12900K multi-core CPU was used for data preprocessing and experimental scheduling. The system was equipped with 64 GB of RAM, which was adequate to enable efficient loading and caching of the complete dataset during training, thereby avoiding frequent disk input/output overhead. The overall hardware configuration did not rely on server-grade or specialized acceleration devices but was instead designed to reflect reproducible and broadly applicable experimental conditions, indirectly demonstrating the potential deployment advantages of the proposed method in resource-constrained scenarios.

The software platform was constructed based on a mature deep learning and scientific computing ecosystem. Ubuntu 22.04 LTS was adopted to ensure environmental consistency and experimental reproducibility. Model construction and training were implemented using PyTorch 2.1.0 with CUDA 11.8 support for automatic differentiation and GPU-accelerated computation, running on Python 3.10. This was used in conjunction with numerical computation and signal processing libraries, specifically NumPy 1.24 and SciPy 1.10, for electromyography data preprocessing, augmentation, and evaluation. A unified random seed (set to 42) was fixed throughout the experiments to reduce the influence of stochastic variation across different experimental runs. The overall software environment fully supported the complete experimental pipeline, from raw electromyography signal processing to model training and performance evaluation.

In the experimental design, identical preprocessing parameters, including a 20–450 Hz fourth-order Butterworth bandpass filter and z-score normalization, were consistently applied across all participants to ensure data uniformity. During segmentation, the sliding-window length was explicitly set to L=250 ms with a stride of S=50 ms. To label overlapping windows containing partial activation, a window was designated as active if more than 50% of its temporal duration overlapped with the manually annotated activation period; otherwise, it was labeled as a resting phase. The original dataset was partitioned into training, validation, and test sets according to a fixed ratio, with 70% of the data used for training, 15% for validation, and 15% for testing, in order to ensure mutual independence among model training, hyperparameter tuning, and performance assessment. The proposed lightweight architecture resulted in a highly efficient model with a total of 24,580 trainable parameters. For the slow-activation-aware temporal attention module, the neighborhood radius was empirically set to k=2. During training, a mini-batch stochastic gradient descent strategy was adopted with the batch size set to B=128. The initial learning rate was set to α=0.001, paired with a Nesterov momentum coefficient of 0.9 and a weight decay term of $1\times 10^{-4}$. The learning rate was adaptively reduced by a factor of 0.5 if the validation loss plateaued for 5 consecutive epochs to balance convergence speed and stability, and an early stopping criterion was triggered if no improvement was observed for 15 epochs. Finally, the decision threshold θ was determined through a grid search ranging from 0.5 to 0.9 on the validation set, yielding an optimal value of θ=0.75. In addition, a strict subject-wise 5-fold cross-validation protocol was employed for overall performance evaluation to prevent potential temporal or subject-specific information leakage. Specifically, the dataset partitioning was performed strictly at the subject level rather than at the window or sample level. The participants were divided into 5 non-overlapping subsets, ensuring that all continuous data trials from a given group of subjects were used exclusively as the validation set, while the data from the remaining subjects were used for training in each fold. Final performance results were obtained by averaging across all folds, thereby providing a realistic estimate of model generalization and better reflecting the expected performance in real-world deployment scenarios.

#### 4.1.2. Baseline Models and Evaluation Metrics

Several representative baseline methods are selected to comprehensively compare different modeling paradigms for the slow muscle activation onset detection task. The traditional fixed threshold method [47] is characterized by a simple structure and extremely low computational overhead, making it suitable for scenarios with stringent real-time requirements. The adaptive threshold method [48] introduces dynamic parameter adjustment on this basis, enabling partial adaptation to varying signal amplitude levels. The sliding-window statistical method [49] characterizes signal variation trends through local statistical features and exhibits good stability under relatively stationary noise conditions. Support vector machine [50] and random forest models [51] based on handcrafted features combine statistical descriptors with nonlinear discriminative capability, thereby effectively improving the separation between activation and non-activation states. The 1D-CNN [52] exploits convolutional structures to extract local temporal patterns and demonstrates strong feature learning ability for time-series signals, while the temporal convolutional network [53] further expands the temporal receptive field through dilated convolutions, which is beneficial for capturing longer-term variation trends. The BiLSTM [54] is capable of modeling both forward and backward temporal dependencies, offering advantages in representing complex temporal dynamics. The Transformer [55], built upon a global self-attention mechanism, explicitly models long-range temporal dependencies and shows strong representational power in fine-grained temporal analysis tasks. To ensure a fair and rigorous comparison, all baseline methods underwent systematic hyperparameter tuning on the validation set. Specifically, for traditional approaches such as threshold methods, optimal parameters were determined via exhaustive grid search. For deep learning models, particularly the Transformer baseline, we explicitly aligned the input sequence lengths with the temporal requirements of the slow activation task and carefully scaled the model capacity, including embedding dimensions and the number of attention heads. This ensures that the Transformer was not disadvantaged by inadequate temporal context or inappropriate parameter choices. Collectively, these baseline methods represent mainstream approaches to slow activation onset detection and temporal modeling from different perspectives, providing a comprehensive reference for comparative evaluation of the proposed method.

The performance of slow muscle activation onset detection was comprehensively evaluated using onset detection error, detection delay, false positive rate, and real-time performance metrics, which respectively characterize temporal accuracy, response speed, stability and reliability, and engineering deployability. Let the manually annotated ground-truth onset time in the *i*-th trial be denoted as $t_{i}^{gt}$, the model-predicted onset time as $t_{i}^{pred}$, and the total number of samples as *N*. The evaluation metrics are defined as

(11) $$\mathrm{ODE}=\frac{1}{N}\sum_{i=1}^{N}\left|t_{i}^{pred}-t_{i}^{gt}\right|,\;\mathrm{Delay}=\frac{1}{N}\sum_{i=1}^{N}\max\left(0,\;t_{i}^{pred}-t_{i}^{gt}\right),$$

(12) $$\mathrm{FPR}=\frac{N_{fp}}{N_{fp}+N_{tn}},\;\mathrm{RTF}=\frac{T_{proc}}{T_{sig}}.$$

Here, ODE denotes the average onset detection error and measures the temporal deviation between predicted and ground-truth onset times. Delay represents the average detection latency, considering only cases where the predicted onset occurs later than the ground truth, thereby reflecting the degree of response lag. $N_{fp}$ and $N_{tn}$ denote the numbers of false positives and true negatives, respectively, and are used to compute the false positive rate FPR. RTF denotes the real-time factor, where $T_{proc}$ is the time required by the model to process the signal and $T_{sig}$ is the actual duration of the corresponding input signal.

### 4.2. Comparison with Different Baseline Methods

This comparative experiment was designed to systematically evaluate the overall performance of the proposed lightweight temporal attention method in the slow muscle activation onset detection task and benchmark it against traditional signal processing approaches, classical machine learning models, and representative deep learning methods. By conducting five-fold cross-validation under a unified dataset and consistent evaluation criteria, the experiment comprehensively characterized the applicability of different methods in slow activation scenarios from four perspectives, including onset localization accuracy, response latency, false positive control, and real-time efficiency.

**Figure 5:**
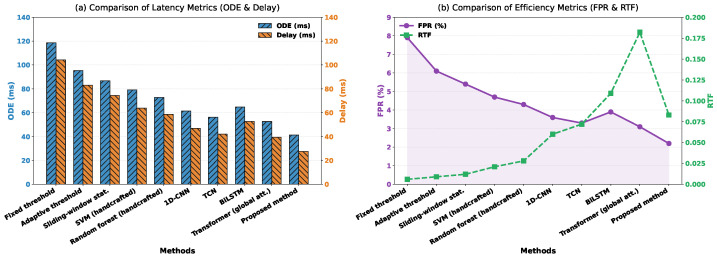
Quantitative performance comparison of the proposed method and various baseline models on the slow muscle activation onset detection task. The plots illustrate the mean and standard deviation of the onset detection error, detection delay, false positive rate (FPR), and real-time factor (RTF) obtained across a five-fold cross-validation scheme.

As shown in Table 3 and Figure 5, traditional threshold-based and statistical methods exhibit clear advantages in real-time performance, yet perform poorly in terms of onset detection error and latency. Under slow activation conditions, their limited capability to model gradual temporal changes results in noticeably delayed onset decisions and relatively high false positive rates. The SVM and random forest models based on handcrafted features provide moderate improvements in detection accuracy and stability, indicating that incorporating statistical descriptors and nonlinear decision boundaries is beneficial for distinguishing activation from non-activation states. However, their performance remains constrained by the representational capacity of manually designed features, which are insufficient to fully capture the continuous and subtle dynamics inherent in slow activation processes.

Among deep learning approaches, 1D-CNN and TCN significantly reduce onset detection error and latency, reflecting the effectiveness of convolutional structures in modeling local temporal patterns. In particular, TCN benefits from dilated convolutions that expand the temporal receptive field, leading to improved onset localization compared to standard CNNs. Nevertheless, these models still rely primarily on fixed convolutional hierarchies for temporal dependency modeling, which limits their ability to represent long-term continuous trends. Although BiLSTM can explicitly model bidirectional temporal dependencies, its recurrent structure is more susceptible to noise accumulation and instability during gradient propagation in slow activation scenarios, resulting in detection latency and false positive rates that are not substantially better than those of convolution-based models. Transformer-based models leveraging global self-attention further improve detection accuracy, demonstrating the value of global temporal relationship modeling for slow activation tasks. However, the uniform modeling of relationships among all time steps introduces a large number of redundant degrees of freedom from a mathematical perspective, making the model prone to assigning non-essential weights to noisy segments and substantially increasing computational overhead. In contrast, the proposed method achieves the best overall performance across all accuracy-related metrics while maintaining acceptable real-time efficiency. This advantage arises from the targeted structural constraints imposed on temporal dependency modeling. By compressing redundant degrees of freedom through lightweight temporal encoding and focusing attention on temporally sustained and informative segments via slow-activation-aware attention, noise interference is reduced while the cumulative effect of gradual changes is enhanced, enabling earlier and more stable onset detection. This balanced design between modeling capacity and structural regularization allows the proposed method to demonstrate clear comprehensive advantages in the high-noise and weak-change context of slow muscle activation onset detection.

### 4.3. Module Ablation Results

The ablation study was designed to quantitatively analyze the functional roles and interactions of the three key modules proposed in this work for the slow muscle activation onset detection task, thereby validating the necessity and synergy of each component within the overall framework. By sequentially removing or replacing the lightweight temporal feature encoding module, the slow-activation-aware temporal attention mechanism, and the noise suppression and stable decision strategy under identical experimental settings and comparing the results with those of the complete model, the specific contributions of each module to detection accuracy, response latency, false positive control, and real-time performance can be clearly observed.

**Figure 6:**
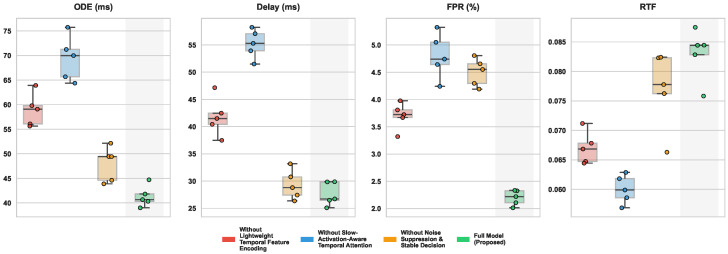
Boxplot distributions of model performance metrics under different architectural ablation settings for the slow muscle activation onset detection task. The plot illustrates the median, interquartile range, and variance of the onset detection error, detection delay, false positive rate (FPR), and real-time factor (RTF) across all cross-validation folds, demonstrating the contribution of each proposed module.

As shown in Table 4 and Figure 6, replacing the lightweight temporal feature encoding module with a simple linear projection leads to a noticeable increase in onset detection error and latency, indicating that shallow convolutional encoding plays a fundamental role in suppressing raw sEMG noise and extracting stable local temporal structures. The most significant performance degradation is observed when the slow-activation-aware temporal attention mechanism is removed, particularly reflected by simultaneous increases in detection error and false positive rate, demonstrating that uniform temporal weighting or the absence of attention is insufficient to distinguish discriminative gradual change segments in slow activation scenarios. When the noise suppression and stable decision strategy is removed, detection latency remains relatively low, but the false positive rate increases markedly, highlighting the critical role of this module in enhancing decision stability and suppressing transient noise interference.

From a theoretical perspective, the ablation results are closely related to the mathematical properties of each module. The lightweight temporal feature encoding compresses redundant degrees of freedom in the input signal through local linear mappings with constrained receptive fields, producing more structured and stationary representations for subsequent modules. When this module is replaced by linear projection, the ability to model local temporal correlations is substantially weakened, making gradual activation trends difficult to capture reliably. The slow-activation-aware temporal attention mechanism imposes structural constraints on the temporal weight distribution, concentrating attention on locally continuous and trend-consistent time regions; its removal causes the model to degenerate toward near-uniform temporal weighting, preventing effective amplification of cumulative slow activation effects and resulting in degraded accuracy and increased latency. The noise suppression and stable decision strategy reduces the degrees of freedom at the decision level by enforcing temporal consistency, thereby limiting sensitivity to short-term anomalies. Without this strategy, the model can still identify potential onset regions at the feature level, but robustness to noise at the decision stage is significantly reduced. The complete model achieves optimal performance across all metrics, indicating that the modules form complementary functions through progressive constraint and information focusing, enabling effective modeling of slow activation onset under weak-change and high-noise conditions.

### 4.4. Generalization Evaluation

The generalization evaluation experiment was designed to assess the stability and applicability of the model under varying signal quality conditions and cross-subject scenarios, thereby examining whether the proposed method remains effective beyond specific experimental settings and can adapt to noise variations and individual differences commonly encountered in real-world applications. By introducing low-noise, medium-noise, and high-noise conditions with different signal-to-noise ratios and further adopting a leave-one-subject-out cross-subject setting, the generalization capability of the model was systematically analyzed from both environmental disturbance and inter-subject variability perspectives.

**Figure 7:**
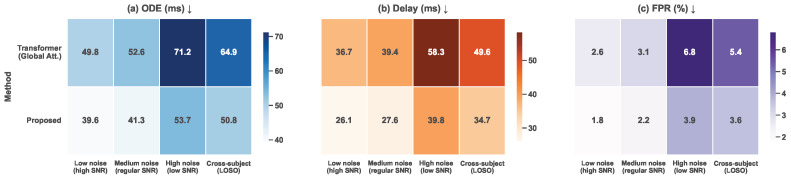
Heatmap visualization comparing the mean onset detection error, detection delay, and false positive rate (FPR) between the proposed lightweight temporal attention method and the global-attention Transformer baseline. The color gradients reflect average performance variations under distinct signal-to-noise ratio (SNR) levels and cross-subject evaluation settings, highlighting the robustness and generalization capabilities of the proposed approach.

As shown in Table 5 and Figure 7, under low-noise conditions, both the Transformer-based baseline and the proposed method achieve favorable detection performance, while the proposed method consistently maintains lower onset detection error, shorter latency, and reduced false positive rate. This indicates that even when signal quality is high, structurally constrained modeling tailored to slow activation characteristics yields more accurate and stable onset localization. As noise levels increase from medium to high, performance degradation is observed for both methods; however, the global attention model exhibits substantially larger deterioration, particularly in detection error and false positive rate, reflecting higher sensitivity to noise perturbations. In contrast, the proposed method maintains a relatively controlled performance decline, demonstrating stronger robustness.

From a theoretical standpoint, the observed differences in generalization performance are closely related to the mathematical modeling characteristics of the respective methods. The Transformer relies on global attention to model relationships among all time steps, which introduces high flexibility in weight assignment. Under noisy or cross-subject conditions, this flexibility allows subject-specific noise patterns and non-informative segments to be incorporated into critical associations, thereby diluting the focus on slow activation trends and increasing false detections and latency. In contrast, the proposed method explicitly restricts temporal dependency modeling through local temporal encoding, slow-activation-aware attention, and stable decision strategies. Attention is concentrated on temporally continuous regions exhibiting gradual trends, while temporal consistency constraints at the decision stage suppress random perturbations induced by noise and individual variability. In cross-subject experiments, these structural constraints further reduce dependence on subject-specific amplitude distributions and noise characteristics, enabling the model to focus on common dynamic evolution patterns inherent to slow muscle activation. Consequently, lower detection error and false positive rates are preserved under LOSO settings, validating the strong generalization potential of the proposed method in practical applications involving diverse users and complex environments.

### 4.5. Discussion

This study focuses on the problem of slow muscle activation onset detection, which has long been overlooked but is of substantial practical value in real-world applications. Both the experimental results and the methodological design indicate that modeling strategies tailored to the characteristics of practical scenarios, namely, slow action initiation, weak signal variation, and unavoidable noise interference, are more meaningful than simply pursuing increasingly complex model structures. In rehabilitation training scenarios, particularly in postoperative rehabilitation, neurological recovery, or exercise training for older adults, patients often find it difficult to perform rapid and explosive muscle contractions. Instead, action initiation typically manifests as low-intensity and gradually increasing voluntary muscle activation. Traditional onset detection methods that rely on fixed thresholds or instantaneous signal changes tend to produce substantial delays in such scenarios, or even fail to trigger reliably. By explicitly modeling the continuity and trend characteristics of slow activation processes, the proposed method is able to identify true onsets earlier and more stably, thereby providing rehabilitation devices with more timely feedback control signals and improving both training safety and interaction naturalness. Rather than merely achieving a numerical reduction in detection latency, the performance of the proposed method fundamentally preserves the integrity of the sensorimotor feedback loop. In clinical rehabilitation, delays exceeding 50 ms can disrupt this loop, leading to patient frustration and uncoordinated movement attempts. By keeping the latency well below this critical physiological threshold, the proposed framework ensures that robotic assistance is closely synchronized with the patient’s initial neural drive, which is considered critical for promoting neural plasticity and accelerating motor function recovery.

In wearable human–machine interaction and assistive control scenarios, slow activation onset detection is equally important. For example, in prosthetic control, exoskeleton assistance, or gesture-based interaction systems, users often express control intent through subtle and gradually increasing muscle activation. If the system responds only when high-amplitude activation is observed, control precision is reduced and interaction may become rigid or even lead to unintended actions. The lightweight temporal attention method proposed in this study enables early responses to such weak activation signals while maintaining real-time performance, allowing the system to better align with natural human motor control patterns. From a biomechanical and mechatronic perspective, the early detection capability of the proposed model provides a crucial time window for mechanical actuators to overcome physical inertia. This precise temporal alignment means that a prosthetic or exoskeleton can begin providing support at the exact moment the user physically initiates the movement, rather than lagging behind. Such synchronization dramatically reduces the metabolic cost of the user and eliminates the rigid “drag” sensation commonly reported in assistive devices. At the same time, the simple model structure and fixed computational pathway make deployment on wearable terminals or embedded controllers practically feasible, facilitating the translation of electromyography-based intelligent interaction technologies from laboratory prototypes to real-world products.

In addition, slow activation onset detection is valuable for long-term motion monitoring and health assessment. In daily activity monitoring of older adults, actions such as standing up or shifting body weight are often accompanied by gradual muscle activation. Accurate identification of these onset moments provides a more reliable temporal reference for fall risk assessment and analysis of motor function decline. The stability demonstrated by the proposed method under varying noise levels and across subjects suggests strong potential for continuous operation and long-term monitoring in complex real environments.

From a computational perspective, the proposed lightweight temporal attention architecture is highly efficient, explicitly addressing the constraints of embedded deployment. The model contains a total of only 24,580 trainable parameters, which is an order of magnitude smaller than standard Transformer baselines that often exceed hundreds of thousands of parameters and significantly lighter than typical 1D-CNN or BiLSTM models used in electromyography analysis. Furthermore, by avoiding the quadratic complexity of global self-attention and the inner-product overhead of conventional local attention variants, the computational complexity of the proposed method scales linearly with the sequence length, denoted as O(T). This drastic reduction in computational burden translates to a significantly lower Real-Time Factor (RTF) of 0.083, compared to 0.182 for the global attention baseline, clearly demonstrating its superior suitability for continuous operation on resource-constrained wearable devices.

### 4.6. Limitations and Future Work

Although the proposed lightweight temporal attention method demonstrates stable and consistent performance in slow muscle activation onset detection, several limitations remain. First, the dataset size and the extent of subject variability remain relatively limited. While the current sample provides a foundational evaluation, testing across a larger and more demographically diverse population is necessary to fully validate the model’s generalizability against broader physiological differences. Second, despite the rigorous inter-rater consensus protocol employed during data labeling, identifying the exact onset of slow, low-amplitude muscle activations inherently carries a degree of annotation uncertainty. The subtle nature of these gradual signal changes makes establishing an absolute ground truth challenging, potentially introducing minor biases into model training and evaluation. Furthermore, while synchronous optical motion data were collected during the experiments, their role in this study was strictly confined to cross-verifying the ground-truth annotations. The current computational framework relies exclusively on single-modality surface electromyography during inference. This unimodal reliance may be insufficient to fully capture motor intent in complex scenarios involving electrode displacement, signal occlusion, or intricate muscle coordination. In addition, the fixed sliding-window segmentation and temporal scale modeling provide practical stability but may not adequately accommodate the substantial variability in slow activation time scales across tasks and individuals. Future work will focus on extending the proposed framework to multimodal settings by integrating inertial, pressure, or visual sensors to enhance robustness, as well as conducting systematic online and long-term evaluations. Furthermore, individual adaptation and few-shot learning strategies may be explored to improve personalization and real-world applicability while preserving the lightweight nature of the model.

## 5. Conclusions

This study addresses the problem of slow muscle activation onset detection, which is frequently encountered in practical applications yet remains insufficiently explored. Motivated by challenges including weak signal variation, long temporal duration, significant noise interference, and constrained deployment conditions, a lightweight temporal attention-based detection framework is proposed for engineering-oriented applications. The method integrates lightweight temporal feature encoding, slow- activation-aware temporal attention, and noise suppression with stable decision strategies, avoiding the computational redundancy of deep architectures and global attention while explicitly enhancing the modeling of critical temporal information during slow activation phases. Experimental results demonstrate that the proposed approach consistently outperforms traditional threshold-based methods, classical machine learning models, and representative deep learning baselines in terms of onset detection error, detection delay, and false positive rate. Higher accuracy, recall, and precision are achieved under regular noise conditions, and stable performance is maintained across different noise levels and subjects. These findings validate the effectiveness, robustness, and deployability of the proposed method, providing a practical solution for slow muscle activation onset detection in wearable interaction, rehabilitation assistance, and long-term motion monitoring scenarios.

## Figures and Tables

**Table 1:** Summary of existing methods and their limitations in slow muscle activation detection.

Method Category	Detection Paradigm	Limitations in Slow Activation
Traditional Threshold & Statistical Methods	Event-based detection relying on abrupt signal amplitude or energy changes.	Highly sensitive to noise; fails to reliably distinguish gradual low-amplitude evolution from background fluctuations.
Standard Deep Learning (CNNs/RNNs)	Data-driven feature extraction via local receptive fields or hidden state propagation.	CNNs lack the receptive field for prolonged trends; RNNs suffer from noise accumulation over extended temporal windows.
Transformers & Global Attention	Global temporal dependency modeling across all time steps.	Introduces computational redundancy; prone to assigning excessive attention weights to noisy, non-informative segments.
Generic Lightweight Models	Structural simplification for embedded classification or regression tasks.	Lacks targeted temporal segment selection mechanisms necessary to isolate weak variations over long durations.

**Table 2:** Statistics of multimodal data for slow activation actions.

Action	Modality	Sampling/Volume
Elbow flexion–extension	sEMG	1000 Hz, 30 × 6
	Video	30 fps, 1920×1080, 180
	Annotation	180 labels
Knee flexion–extension	sEMG	1200 Hz, 28 × 6
	Video	25 fps, 1920×1080, 168
	Annotation	168 labels
Shoulder abduction/adduction	sEMG	1000 Hz, 30 × 5
	Video	30 fps, 1280×720, 150
	Annotation	150 labels
Standing center-of-mass shift	sEMG	800 Hz, 26 × 5
	Video	20 fps, 1920×1080, 130
	Annotation	130 labels

**Table 3:** Comparison results with different baseline methods on the slow muscle activation onset detection task (five-fold cross-validation average ± standard deviation).

Method	ODE ↓ (ms)	Delay ↓ (ms)	FPR ↓ (%)	RTF ↓
Fixed threshold method	118.6 ± 14.2	104.3 ± 12.8	7.9 ± 1.1	0.006 ± 0.001
Adaptive threshold method	95.4 ± 11.5	83.1 ± 10.4	6.1 ± 0.9	0.009 ± 0.002
Sliding-window statistical method	86.7 ± 10.1	74.5 ± 9.2	5.4 ± 0.7	0.012 ± 0.002
SVM (handcrafted features)	79.2 ± 8.4	63.8 ± 7.6	4.7 ± 0.6	0.021 ± 0.003
Random forest (handcrafted features)	72.8 ± 7.9	58.6 ± 6.8	4.3 ± 0.5	0.028 ± 0.004
1D-CNN	61.5 ± 5.8	46.9 ± 5.1	3.6 ± 0.4	0.060 ± 0.005
TCN	56.2 ± 5.3	42.1 ± 4.7	3.3 ± 0.4	0.072 ± 0.006
BiLSTM	64.8 ± 6.5	52.7 ± 5.9	3.9 ± 0.5	0.109 ± 0.008
Transformer (global attention)	52.6 ± 4.9	39.4 ± 4.2	3.1 ± 0.3	0.182 ± 0.012
Proposed method (lightweight temporal attention)	41.3 ± 3.5	27.6 ± 2.8	2.2 ± 0.2	0.083 ± 0.007

**Table 4:** Module ablation results (five-fold cross-validation average ± standard deviation).

Configuration	ODE ↓ (ms)	Delay ↓ (ms)	FPR ↓ (%)	RTF ↓
Without lightweight temporal feature encoding	58.9 ± 5.4	41.8 ± 4.5	3.7 ± 0.4	0.067 ± 0.005
Without slow-activation-aware temporal attention	69.4 ± 6.8	55.2 ± 5.7	4.8 ± 0.6	0.060 ± 0.004
Without noise suppression and stable decision	47.9 ± 4.6	29.3 ± 3.1	4.5 ± 0.5	0.077 ± 0.006
Full model	41.3 ± 3.5	27.6 ± 2.8	2.2 ± 0.2	0.083 ± 0.007

**Table 5:** Robustness and cross-subject generalization results compared with the global attention baseline (average ± standard deviation).

Experimental Setting	Method	ODE ↓ (ms)	Delay ↓ (ms)	FPR ↓ (%)
Low noise (high SNR)	Transformer (global attention)	49.8 ± 4.2	36.7 ± 3.8	2.6 ± 0.3
Low noise (high SNR)	Proposed method	39.6 ± 3.1	26.1 ± 2.5	1.8 ± 0.2
Medium noise (regular SNR)	Transformer (global attention)	52.6 ± 4.9	39.4 ± 4.2	3.1 ± 0.3
Medium noise (regular SNR)	Proposed method	41.3 ± 3.5	27.6 ± 2.8	2.2 ± 0.2
High noise (low SNR)	Transformer (global attention)	71.2 ± 8.5	58.3 ± 7.4	6.8 ± 0.9
High noise (low SNR)	Proposed method	53.7 ± 5.6	39.8 ± 4.5	3.9 ± 0.5
Cross-subject (LOSO)	Transformer (global attention)	64.9 ± 7.2	49.6 ± 6.1	5.4 ± 0.7
Cross-subject (LOSO)	Proposed method	50.8 ± 4.8	34.7 ± 3.9	3.6 ± 0.4

## Data Availability

The data presented in this study are available upon request from the corresponding author.
